# Medical Sketches Taken in the Royal Navy

**Published:** 1812-04

**Authors:** James Robinson Scott

**Affiliations:** SURGEON, Royal Navy


*For the Medical and Physical Journal.*

### MEDICAL SKETCHES *taken in the* ROYAL NAVY, *by* JAMES ROBINSON SCOTT, SURGEON, R. N.

(Continued from p. 195.)

### *On Depletion with the Lancet, and the use of Opium.*

WERE I to enter into detail, I could produce numerous cases where the most extensive direct depletion has been used for the cure of pneumonia; where not only no subsequent injury occurred from its use, but where, if it had been neglected, the most fatal effects might have arisen. I shall give the outline of two cases which lately fell under my observation, and the reflections to which they gave rise.

In December last a patient applied to me with every evident symptom of inflamed lungs: he was considerably above 40 years of age, and of a spare habit. I immediately bled him to the extent of 25 ounces, in a moderate stream; he did not feel immediate debility; his pain was then acute, and his breathing very difficult; he had labored under the complaint before. I added to this depletion, a laxative and antimonials. Next day he was much better—pain greatly abated; but the day after this he got weak, had nausea, foul tongue, pain in the head, &c. He was then breathing impure air, and perhaps had possessed a strong susceptibility of the effects of miasmata. I did not again bleed him; his pain in the breast had ceased, but there followed certainly a fever bearing a similitude to that species commonly called typhoid. He was speedily removed to good air, and diet suited to his situation, where he soon recovered. I may ask—did his debility come on merely because he lost at first 25 ounces of blood, or because he was subjected to air not altogether pure, or both these together? Did these typhoid symptoms necessarily necessarily follow his depletion? Were they to be expected from that treatment? Was not the danger of pulmonary inflammation proceeding to its fatal acme, greater than the chance of some temporary debility being produced? Had V.S. been neglected, or triflingly put in force, this effect was to be expected with considerable certainty. The patient recovered, and the danger of pneumonia was averted.

I was forcibly struck with the situation of a patient whom I saw lately, almost expiring under oppression of the præcordia. He was a young man, had been often afflicted with pulmonary complaints, for which direct depletion was used with effect. His present illness had lasted eleven days. When I saw him his pulse was 110 and rather full; he expectorated much frothy mucus, had an incessant pain in his right breast, and inspired with severe struggles. He had been ill for eleven days, was very weak, and, as he was to be sent to an hospital next morning, I did not use the lancet. Being ignorant of the circumstances attending the progress of his disease, except as related by himself, I concluded likewise that if pneumonia had been going on for this length of time, effusion of some sort had taken place. Indeed bleeding, after six days duration of the complaint, is not often effectual; for by this time, what is improperly called "*vis medicatrix naturæ*," has generally done its utmost: the inflamed viscus is nearly exhausted, inability to perform the circulating action completely deranged, and a dreadful state of indirect debility is then mostly present. This reflection, and an apprehension that convulsive asthma was present, induced me to give him a composing and diaphoretic draught for the night. Next day he was sent to the hospital. He afterwards lost blood there to the amount of upwards of 50 ounces at different bleedings, but died some days subsequent to his admission. On inspection, *post mortem*, I am informed that hydrothorax was obvious. This man was invalided before his last illness for a state of respiration, the effect of reiterated pulmonary inflammation. The effusion into the cavity of the breast I conceive to have been caused by his last attack, or previously, by some similar cause; and, as it was perhaps difficult to determine whether incipient hydrothorax had been existing prior to, or at the time he was seized with, fatal illness, I am decidedly of opinion that the best reasons existed for bleeding this patient largely and promptly at its commencement. The physicians, by ordering V.S. even at the advanced stage, prove that their experience and practice were most judiciously exercised; for if effusion had not taken place, a chance, and the only one existing, was offered for life.

In

In His Majesty's convalescent ship Gladiator, in which I served for some time as assistant surgeon, I had frequent views of the state of patients sent there to be afterwards invalided. Of these the majority labored under complaints the effect of pulmonary inflammation. Almost all experienced, where it had been employed, the good effects of blood-letting at different times: but in my conversations with these men about their previous complaints, this evacuation, I often found, had been neglected, or perhaps purposely laid aside, to the manifest injury of the sufferer under this chronic misery.

Next to thoracic inflammation, as connected with depletion, that disease which demands the consideration of naval medical practitioners, is rheumatism. Opinion has been much divided about the propriety of taking away large quantities of blood in this excruciating complaint. It would be a happy circumstance, indeed, if a decided plan of treatment, founded on the best experience, and more capable of alleviating the sufferings of sailors attacked with this complaint, than those existing at present, was established. Of all other remedies, opium bids fairest to acquire the title of specific, in this disease. The precise nature of rheumatism is not at present fully known. We know, however, with certainty, that pyrexia is present; that the nervous system is much deranged, and extremely susceptible of pain; that this great degree of pain, whether it may be solely the consequence of inflammation or not, proves a continued exciting cause to the disease, and generates more pain. The irritation referable to nerves is here so obvious, and re-acts on the vascular system so much, that the absence of it must of all things facilitate recovery. Allowing that this pain is caused by inflammation in the great joints, in the fasciæ, or in the muscular fibre, yet so much nervous sensibility exists, that, by lessening with opium the susceptibility of the sensorium to receive these painful impressions, and the nerves to transmit them, we gain ground and cut off one great source of irritation. If by depletion we could avert danger in the same ratio as we can in thoracic inflammation, then the use of opium might be better dispensed with: but direct depletion is by no means, however often repeated, adequate to the removal of acute rheumatism; and, were we in this disease to be guided by the appearance of the blood, we might bleed *ad infinitum* without altering that appearance, or removing the disease. The effect of opium here, in allaying pain, much more than compensates for its previous stimulating action; but the doses should be large, sufficient to produce a speedy state of torpor: the state of the system then demands it. There is not that danger threatened in rheumatism by vascular distention, as in thoracic inflammation: the parts attacked are not so necessary to vital action. Where I have bled in rheumatism, or have seen others do so, I have also used opiates in conjunction, and seen them used by others. But I have treated rheumatism with opiates and did not bleed, and with the best effect. And here I must advert to the successful method of treating acute rheumatism, with opium, practised by Dr. George Pearson, my scientific and enlightened preceptor, where so much evidence results from the doctor's experience, as to prove its superior utility incontestible. Many practitioners of the navy corroborate the truth of the doctor's principles in this case, and since I joined the service I have seen every thing to induce me to be a warm advocate for the use of opium in this disease.

From many cases which I could adduce of the utility of opiates in rheumatism, I shall give one in which, though every cause inimical to recovery surrounded the patient, the greatest benefit resulted from the plan of treatment pursued.

Henry Tarr, landsman, aged 22 years, taken ill at Lisbon, May 5, 1810, complains of severe wandering pains in his shoulders, breast, and extremities; bowels irregular; skin hot; tongue foul; not much thirst; pulse 110.—N. B. He has injured his constitution much by drinking. Grog and wine entirely prohibited.

℞. Pulv. convolv. jalapæ ℈j.
Submuriat. Hydrarg. gr. v.
M. ut. F. Bol. stat. sumend.

℞. Oxid. Antimon. c. phosphat. calcis. gr. v.
ft. bolus, h. s. s.

6th. Much the same as yesterday; contin. med. antimon. Purgative has operated briskly; diaphoresis. Pains continue; tongue foul; the antiphlogistic regimen enforced, and to dilute largely. 8th. Pains increase in severity, numbness in the extremities, and cramp. Contin. medicamentum antimoniale; pediluvium used, and flannel applied round all the joints. 9th. Very weak; tongue foul; pulse above 100; dreadful pain in the wrists and feet. Bol. purgans ut antea. repet. Contin. med. ant. h. s. 10th. Perspiration free and plentiful; great debility; loss of appetite; purgative has operated briskly; restless and sleepless night. ℞. Pulv. ipecac. c opio. gr. xv. h.s.s. 11th. Better sleep last night; tongue still foul; perspiration free. Contin. med. opiat. 12th. Pulse not lessened; tongue still foul; slept better last night, and wishes the continuance of his powder.

13th.

13th. Bowels regular; tongue cleaner. 14th. Pains still continue; pediluv. tepid; pulse about 100. V.S. ad ℥xii. No appearance of buffy coat. cont. med. 15th. Tongue cleaner; appetite better; pulse 90; perspiration free; heat of skin abated; diet to be light and more nourishing; pains over the whole of the lower extremities, and severe. Contin. pulv. ipecac. c opio. 16th. Great debility; tongue cleaner. Repr. bol. purgans. ℞. Pulv. ipecac. c opio. gr. xv.

Opii pur. gr. ij. M. ut Ft. bol. h.s.s. 17th. Purgative has produced a number of large stools; pediluvium tepid. Repet. medicamenta opiata h. s. Tongue clean; pulse varies from 90 to 110; heat of skin much abated. 18th. Pulse 90, and soft; thirst much lessened. 19th. Much better every way. Cont. opiata. 20th. Feels light and easy; appetite better. 21st. Difficulty in moving, from stiffness in the joints, but is much better; sat up to-day for some time. ℞. Opii pur. gr. ij. h.s.s. Cont. pediluvium. 22d. Ordered to sit on deck for some time in the sun. 23d. Back sore and stiff. Cont. opiat. h. s. 24th. The same. 25th. Better; pains variable. Cont. opiat. 26th. The same. 27th. Cont. opiat. Better every way. 28th. Rep. bol. purgans. Cont. opiat. 29th. Purgative operated. 30th. Convalescent; some exercise enjoined; no wine allowed yet; his appetite is good; he is recovering strength and a freshness of color; opiates discontinued; bowels to be kept regular.

June 20th, 1810. Having arrived at Plymouth, I think it adviseable to send him to the hospital. We have been at sea during his illness, where the moist and bad atmosphere between decks has retarded his recovery. The removal of acute rheumatism, in a vessel such as this, is both difficult and uncertain. A custom which prevails here of deluging the decks with water every morning, has been very injurious in this case, as well as in many others. I endeavored as much as possible to prevent this custom, but was not successful owing to the commander's obstinacy.

Had I a similar case to treat again, under similar circumstances, I should give more opium than I did in this, and bleed at the commencement of the disease. This patient had a cachectic habit, little strength, and much irritability. I purged him briskly throughout his illness, and his debility did not prevent me. He was at first costive, and the opium given to him required this to be guarded against. He was always better after being purged freely. I continued this plan until his tongue got clean. I think that Dr. Hamilton's invaluable practice* was applicable in this case. His mouth

* Vide Hamilton on Purgatives.

was was not affected by the calomel. He experienced the greatest relief from the opium and Dover's powder; and, although his perspiration was free from the beginning, increased by the antimonials administered, yet his pains were uncommonly severe until I gave him the opium. When pulv. ipecac. c opio. is given in cases of acute rheumatism, Dr. George Pearson attributes the good effects produced by it entirely to the opium it contains; and I think with the greatest propriety. There was no buffy coat on this patient's blood. I was deterred from using direct depletion here to any extent, on account of the patient's constitution, and the great degree of debility, tending to typhus, which appeared, although this did not, as I mentioned before, prevent me from purging largely; and this, with the regimen enjoined, kept him sufficiently low. Had there been any pulmonary affection present, any tendency of the lungs to become inflamed, any pain in the thorax, I should have used direct depletion freely, notwithstanding the other symptoms; but, as this was not the case, and as I did not perceive the advantage of bleeding in his precise situation, I did not evacuate by the lancet.

Dr. Pearson does not, in his practice, find that bleeding in acute rheumatism is of much service; he puts the greatest faith in opium, and his success is proportionate to this opinion of the medicine. Perhaps, however, what is proper in the practice of a physician in London, will not, even in this disease, hold good a-board ship, and at sea. As I mentioned before, inflammatory complaints attack sailors with peculiar violence, from the magnitude of the exciting causes to which they are exposed. The vigor of their constitutions also enables them to bear direct depletion with more effect than those subjected to the luxuries of a metropolis. Dr. P. would always, however, bleed freely where there was a chance of the lungs becoming affected.

I shall conclude these remarks on the propriety of large direct depletion in rheumatism, by stating my opinion of the most rational plan of treating this excruciating disease on board of his Majesty's ships, and preventing subsequent bad effects from taking place, as far as actual observation and reflection enables me to give this opinion.

1st. If the patient be not above 40, and in any way plethoric, in the very first instance, 20 or 25 ounces of blood taken away will answer an excellent purpose: it may prevent much mischief in case a determination should take place to any viscus, and can do no harm at all events. 2d. Opium in large doses ought to be administered. the degree of pain experienced by the patient will be the best guide to the the repetition of the opiates. 3d. Flannel cloathing, and a dry situation, are indispensable. 4th. Antimonials, alternating with the opiates, or conjoined with them. 5th. Bowels kept clear by occasional brisk purgatives. 6th. Diluting beverage and low regimen. 7th. Blistering and sinapisms. 8th. If the patient has any obvious predisposition to phthisis, or if he has had any attack of thoracic inflammation, bleeding ought not to be dispensed with, but it should be accompanied with opiates. 9th. If, after bleeding the patient once or twice freely, according to circumstances, he should feel in no degree relieved, then opium is chiefly to be trusted to.

I have very little faith in external applications in this disease. Some practitioners extol highly the use of bark taken internally, and others give great praise to ol. terebinthinæ used in a similar way. Of these medicines I have had, personally, no experience; and shall decline offering any observations upon them.

I cannot quit this subject without mentioning the particulars of a singular case of acute rheumatism which I treated in 1810, and which terminated in an effusion of lymph or serum into the knee-joint, thickened the ligaments by pressure, and nearly destroyed the use of the limb. The patient's constitution had been broken by intemperance: he stated his age as thirty-one years, but he appeared to be upwards of forty. On being seized with the disease, a most unusual degree of debility took place; entire loss of appetite; tongue much furred; pulse about 110, and weak. In a few days the knee-joint swelled rapidly, and fluctuation soon was discoverable. He had no rigors; no pointing of matter to any particular spot; nor any symptoms intimating the existence of phlegmonous inflammation in the part. I took him, with his knee in this state, to the hospital-ship in the river Tagus. The medical officer there, who received him from me, was of opinion that phlegmonous inflammation had existed; and, as I had not bled my patient, he seemed to insinuate that the proper antiphlogistic treatment had not been promptly enough applied, and even that I had mistaken the nature of the local affection. My applications to the joint, after the swelling appeared, were mercurial frictions, to promote absorption; previously I had used the saturnine lotion. A consultation was afterwards held on this case: the result was that pus had not formed. No opening was made into the swelling; saturnine lotion was continued for some time; the leg became very œdematous, but the effused fluid was afterwards absorbed, and he recovered, but not the full use of his limb, the power of flexion being partly destroyed. I subsequently found that the patient had been using spirits clandestinely while under my care. I regretted that I had not used direct depletion, although I am disposed to think that it would not, in this individual case, have been attended with much success. The patient's constitution had been broken down by excess, and there was great debility. But, where the subject is healthy and sanguineous, copious bleeding may in many instances prevent a local affection, serious in its nature, or stop its progress; but in this case the swelling came on with uncommon rapidity.

The next disease which strongly calls for promptitude of treatment, belonging to the class Pyrexiæ, and which so often occurs on-board ship, is Dysentery. Direct depletion has been found a most excellent auxiliary in its cure, and, if used at all, should not be delayed after the symptoms have become manifest; particularly if the patients be young and robust. That important balance of circulation which exists between the skin and different viscera, and which I adverted to when giving an outline of the theory of thoracic inflammation in the beginning of this sketch, is much sooner restored, and the cause of the disease removed, in patients of the above description, when bleeding is freely used in the first instance: indeed I question whether, on considering the pathology of the intestinal canal, and seeing this practice supported with great success in the navy by many of its most enlightened and judicious physicians, we ought not almost to consider venesection as the grand indication of cure. Among those practitioners in the navy who favor the use of the lancet in dysentery, there is one whose knowledge of its utility is founded on extensive observation, as his arguments for it are supported by the most rational philosophy,—a gentleman well known to all the naval medical establishment as a learned and discriminating physician. His words are nearly to this effect, when speaking of dysentery:

"If you *bleed early* in this disease, you will greatly assist your intentions of restoring cuticular perspiration ; you will determine from the intestinal canal, reduce the vascular action, the balance of circulation will be much more speedily recovered than if bleeding be neglected."

On taking a view of this disease, as it occurs on-board ship, powerful reasons will arise for bleeding in conjunction with other means of cure ascertained by general experience to be useful.

The dysenteric contagion is specific. It appears to be either produced originally in the living body, or by some unknown compositions or decompositions which occur in the egesta of those whose intestines are in a peculiar morbid state.

The remote or predisposing cause of this formidable complaint, appears to be a peculiar state of the intestinal canal, produced by the unwholesome stimulus of certain ingesta for a given time; at sea most generally by fluid substances, such as bad water on a long voyage. In a warm country, water which has been kept in casks not properly sweet, sour wine, water which has been put into beer-casks, &c. seem peculiarly noxious.

The intestines thus predisposed to disease, it becomes excited by exposure to cold, wet, &c. or to any cause which will check the action of perspiration, and destroy that important balance of circulation existing between the surface of the body and different important viscera. The blood thus determined to the intestines already predisposed to disease, effusion from the extreme mesenteric arteries, which terminate upon the villous coat of the intestines, takes place; the conjoint effect of vascular distention and irritation in the membrane.

The symptomatic fever which exists in dysentery, but which does not characterise the disease, appears to be just such a one as accompanies a severe inflammatory affection of any important viscus: its cause is suspended perspiration and local irritation. This fever does not appear to be specific, although we have great reason to suppose that the local affection which helps to produce it is. If we bleed, and thus lessen inflammation, while we endeavor to restore perspiration and lessen irritation in the intestines, we subdue local disease, and consequently the fever subsides.

We have every reason to believe that this disease is entirely distinct in its nature from that which is called typhus. The contagion appears to be *sui generis*. When once the disease has begun, the determination of blood to the intestinal tube, the peculiar inflammatory action in the vessels terminating on the mucous membrane, and the deranged state of its secretions, are the symptoms most alarming. They call for a speedy removal, for the danger of a fatal termination is in proportion to the length of their continuance. Bleeding in moderation holds forth a prospect of removing those symptoms speedily. The irritation referable to nerves in the intestines will be best lessened by opium, which of all other medicines is the most useful in dysentery.

Although great debility must necessarily exist in dysentery, this ought not, no more than in pneumonia, to withhold a practitioner from bleeding at the commencement of the disease; for is it not the effect of a peculiar inflammatory action, rendering an important viscus unfit to perform its functions? While this inflammation lasts, the whole chylopoietic poietic viscera are deranged, and the actions of digestion, &c. either partially or wholly suspended. We perceive the cause of all this to exist, on the one hand in determination to the intestines, and on the other in the action of a morbid stimulus. Lessen this determination to the intestines by restoring perspiration, which bleeding will greatly assist in, (and also by bleeding give the distended vessels ease,) and you will subdue inflammation. Diminish by opium the high degree of sensibility and irritability that exists in the intestines, and the morbid stimulus will cease to act with its wonted violence; and it will soon, by the application of proper additional means, such as ventilation, cleanliness, &c. cease to reproduce itself.

As in rheumatism, the effects of opium in this disease, by its lessening morbid sensibility, more than counterbalances its stimulating properties; and, if before fulfilling our intentions in its exhibition, some increase of circulation be produced, there is not that imminent danger which attends pneumonia. Effusion of blood is not a fatal consequence of dysentery; it is of pneumonia. In fact, the least chance of giving additional distention to the blood-vessels, or of increasing the rapidity of circulation when the lungs are inflamed, must be avoided with the utmost circumspection.

Opium in this disease, proves useful chiefly by its action on the internal or villous coat of the intestine. Calomel is in high request with many practitioners. I do not conceive how the latter can be used with any rational prospect of success, unless given with the design of determining suddenly and largely to the salivary glands, when the disease continues obstinate. In this case it should be combined with opium, to prevent its further irritating the intestine, already stimulated beyond its powers. To exhibit it as a purgative, every rational practitioner will condemn: it is only adding stimulus to stimulus, and when the bowels are in such an irritable state, this is the height of impropriety. A surgeon of the staff, now in Portugal, has been long in the habit of using opium as his only medicine in dysentery; and with the most ample proofs of its efficacy. He succeeds better with the cases which fall under his hands, than others who use calomel either by itself or combined with opium. This was communicated to me when in Lisbon, at a time when the troops had been affected with dysentery. He was going to his post, and had provided himself with some thousand opium pills to administer to his patients in this complaint.

The strongest argument which can be used against bleeding freely in dysentery is, " that the abstraction of such an important important stimulus as the blood in any considerable quantity when the body is brought by the disease into a state of debility, must render it less able to hold out against the complaint for any length of time; particularly as the nutritive faculties are so much deranged." But to this it may be answered, that this debility is more apparent than real; it arises more from the loss of healthy action in the stomach and intestines for a time, than from other causes: and this loss, or derangement of healthy action, is the immediate effect of inflammatory action in the intestine, accompanied by bloody effusion. The febrile weakness existing is that of synocha, not of typhus. Where there has been little exhaustion of excitability, where there is really no typhoid contagion acting on the brain, and affecting the sources of the vital principle, bleeding will be found to restore strength by lessening local inflammatory action, and consequently the constitutional irritation depending thereon.

As far then as can be judged from this view of dysentery as occurring on-board ship, and the practice of eminent naval physicians successful in its cure, the following mode of treatment may be rationally presumed as most applicable, generally speaking, in the navy; it being premised, that variation of constitution and circumstances will induce changes in the treatment, founded on propriety.

1st. On the accession of the disease, warm clothing of flannel to be immediately put on. 2d. The patient put into his hammock, and to lie between blankets. 3d. On the febrile symptoms becoming manifest, let 16 ounces of blood, more or less, according to the habit of body and the age, be taken from the arm in a moderate stream; if a robust and sanguineous constitution, 24 ounces will not be too much. The bleeding to be repeated after the interval of a day, or two days, according to circumstances. 4th. An enema to evacuate the rectum. At first a purgative one for this purpose, and afterwards, according to the severity of the disease, opiate enemas, repeated occasionally, formed with mild emollient substances. 5th. Pills of opium of 1½ grains each, given to the amount of one at bed time, or two in the day, or three, according to circumstances. Antimonial powder to be given either alternating with, or conjoined to, the opiates, in doses of 3 grains, three or four times in 24 hours. Perhaps the best way of giving the opium is in pills, or, if pulv. ipecac. c opio be found to agree well with the patient, it may supersede the antimonials. 6th. The diet to be light and emollient—rice—sago—portable soup, &c. plenty of tepid diluting drink to be allowed. 7th. A dry airy situation for the patient if possible, and the most punctual observance servance of cleanliness; the place around him to be constantly fumigated and sprinkled with vinegar. If, after the first or second bleeding, the patient feels general relief, it is to be repeated according to the practitioner's judgment. If the opiate enema are eminently useful, opium by the mouth to be lessened, and *vice versa*.

